# Correlation of Central Corneal Thickness and Keratometry Findings With the Severity of Diabetic Retinopathy and Glycosylated Hemoglobin (HbA1c) Levels in Type II Diabetes Mellitus Patients

**DOI:** 10.7759/cureus.74116

**Published:** 2024-11-20

**Authors:** Shifa Yousuf, Shovna Dash, Pallavi Sahu, Soumyakanta Mohanty

**Affiliations:** 1 Ophthalmology, Kalinga Institute of Medical Sciences, Bhubaneswar, Bhubaneswar, IND

**Keywords:** central corneal thickness, diabetic retinopathy, glycosylated haemoglobin, keratometry, type ii diabetes

## Abstract

Introduction: Diabetes today is a global health issue, posing a risk to several organ systems. Besides complications like cataracts, diabetic retinopathy (DR), glaucoma and refractive errors, anatomical parameters like central corneal thickness (CCT), which is a crucial indicator of corneal endothelium function and keratometry parameters, have also been noticed to be altered in diabetes. Variations in these parameters may affect the accuracy of applanation tonometry in measuring intraocular pressure. This study aims to investigate variations in CCT and keratometry across different stages of DR, as prior research has yielded inconsistent results. The study also intends to explore the relationship between glycosylated hemoglobin (HbA1C) levels and changes in keratometry and CCT.

Materials and methods: This observational cross-sectional study was conducted from July 2022 to June 2024. A total of 204 subjects, aged 35-70 years, were included in the study. All participants were evaluated for DR, and those showing fundus findings were graded into five classes based on the Early Treatment Diabetic Retinopathy Study (ETDRS) classification. CCT, keratometry parameters (K1 and K2) and HbA1C levels were measured for all participants. The clinical parameters were correlated across different grades of diabetes mellitus and compared using Statistical Package for the Social Sciences (IBM SPSS Statistics for Windows, IBM Corp., Version 23.0, Armonk, NY) software.

Results: The patient population consisted of 130 males (63.7%) and 74 females (36.3%). It was found that there was a trend indicating that the presence of DR increased with age, which was statistically significant (p=0.036). However, the differences in the mean duration of diabetes across DR groups were not statistically significant. Findings suggested that individuals with DR generally possess a higher CCT compared to the control group, particularly in the earlier stages of the disease, i.e. no DR and mild non-proliferative diabetic retinopathy (NPDR). However, as the severity of DR increases, the CCT tends to decrease slightly in moderate NPDR, severe NPDR, and proliferative diabetic retinopathy (PDR) stages. The study notes that the variations in CCT across these groups were not statistically significant (p-value of 0.29). The study also found that changes in corneal curvature, as indicated by keratometry values, were more pronounced in the proliferative stage of DR, with a statistically significant increase in k2 values of the left eye (p-value = 0.025). The study concluded that there was no statistically significant relationship between CCT and keratometry values of both eyes with HbA1c levels.

Conclusion: While there were some clear patterns, especially concerning age, most variations in ocular parameters were not statistically significant, suggesting that other factors might need an in-depth study in the development of DR and its correlation with ocular parameters. Similarly, while HbA1c has often been stated to be a prognostic factor in diabetes mellitus, significant correlations were neither noted with ocular parameters like CCT and keratometry, nor the stage of retinopathy.

## Introduction

Diabetes mellitus (DM) is a significant global health issue, with its prevalence rising sharply in recent years, especially in India [1]. Recent national statistics reveal that diabetes is more prevalent in urban areas, affecting 28% of the population, compared to just 5% in rural regions [2]. The condition is marked by elevated blood sugar levels, leading to vascular alterations that result in structural and functional bodily changes. Common ocular complications of diabetes include cataracts, retinopathy, refractive changes, and glaucoma. Patients often experience not only diabetic retinopathy (DR) but also damage to the corneal endothelium. Consequently, DM influences both keratometry and central corneal thickness (CCT), a crucial indicator of corneal endothelium function [3]. Elevated blood sugar levels alter the endothelium, resulting in corneal edema and increased thickness [4]. Abah et al. found in their study that CCT affects the accuracy of intraocular pressure (IOP) measurements obtained through applanation tonometry [5]. This study aims to investigate variations in CCT and keratometry across different stages of DR, as prior research has yielded inconsistent results. Glycosylated hemoglobin (HbA1C) serves as a long-term indicator of diabetes control, reflecting average blood sugar levels over the previous three months and providing valuable prognostic information. Therefore, we also intend to explore the relationship between HbA1C levels and changes in keratometry and CCT.

## Materials and methods

This prospective observational cross-sectional study was carried out in the Ophthalmology Department at Pradyumna Bal Memorial Hospital, Kalinga Institute of Medical Sciences (KIMS) in Bhubaneswar from July 2022 to June 2024, following approval from the Institutional Ethics Committee (KIIT/KIMS/IEC/955/2022). Patients who visited our outpatient department and those referred from other departments were assessed based on specific inclusion and exclusion criteria. A total of 204 subjects participated in this study.

Inclusion and exclusion criteria

Inclusion criteria included patients aged between 35 and 70 years. Cases were those with type II DM, either on anti-diabetic medications or recently diagnosed as per American Diabetes Association guidelines, and who have the same grade of DR in both eyes. The control group included subjects without DM. Exclusion criteria included patients with significant media opacities that hinder fundus examination, glaucoma, history of trauma, history of ocular surgery including laser refractive surgery, contact lens users, pregnant individuals, and those with local or systemic diseases affecting the cornea, such as dystrophies, degeneration, opacity, keratoconus, or pterygium.

Primary objectives include measuring CCT in patients with type II diabetes and correlating these findings with the severity of DR within the study group, as well as relating keratometry parameters to the severity of DR. Secondary objectives involve assessing the relationship between CCT and keratometry with HbA1C levels.

Study procedure

Informed written consent was obtained from all participants. A comprehensive ocular and medical history was recorded. An extensive ophthalmological examination was conducted, with Best Corrected Visual Acuity (BCVA) assessed for distance and near using Snellen’s chart. IOP was measured using Goldmann applanation tonometry. A slit lamp examination of both the anterior and posterior segments was performed. CCT and keratometry measurements (K1 and K2) were taken with the ZEISS IOLMaster 700 (Carl Zeiss Meditec AG, Jena, Germany). Pupils were dilated with topical medications, including 1% tropicamide and 5% phenylephrine, except for hypertensive patients, where just 1% tropicamide was used. Detailed fundoscopy was conducted using direct and indirect ophthalmoscopy, along with slit lamp biomicroscopy using a 90D Volk lens (Volk Optical, Inc., Mentor, USA). Optical coherence tomography (OCT) and fundus fluorescein angiography (FFA) were performed as needed. All participants were evaluated for DR, with those showing fundus findings graded into five classes based on the Early Treatment Diabetic Retinopathy Study (ETDRS) classification: No DR, mild non-proliferative diabetic retinopathy (NPDR), moderate NPDR, severe NPDR, PDR. An equal number of participants in each class were included in the study.

Laboratory investigations

Fasting blood glucose, postprandial blood glucose, and HbA1C levels were measured for all participants. All the tests were carried out in the morning.

Statistical analysis

Statistical Package for the Social Sciences (IBM SPSS Statistics for Windows, IBM Corp., Version 23.0, Armonk, NY) was utilized for data analysis. Descriptive statistics were provided as means and standard deviations for continuous variables, and frequencies and percentages for categorical variables. The chi-square test was employed to compare categorical data for significance, and one-way analysis of variance (ANOVA) was applied to assess clinical parameters across different grades of DM, with a p-value of less than 0.05 considered statistically significant.

## Results

The patient population comprised 130 males (63.7%) and 74 females (36.3%). The study found that males were more prevalent in the data compared to females, with a male-to-female ratio of 1.7:1. The duration of diabetes ranged from 1 to 140 months, with longer durations associated with the presence of any form of DR. Patients with moderate NPDR had the longest mean duration of diabetes. However, the differences in mean duration across DR groups were not statistically significant. The mean age of patients without DR was 50.6 years, while those with mild NPDR were 55.74 years, and those with moderate and severe NPDR were 55.24 years. The mean age of patients with PDR was 52 years. A trend indicated that participants with DR tend to be older compared to those without retinopathy or those in the control group, which was statistically significant (Table 1).

**Table 1:** Relationship between grades of diabetic retinopathy and age DR: diabetic retinopathy; NPDR: non-proliferative diabetic retinopathy; PDR: proliferative diabetic retinopathy

Stages of Diabetic Retinopathy in Both Eyes	Mean Age	Std. Deviation	P-value
No DR	52.00	8.11	0.036
Mild NPDR	55.74	9.64	
Moderate NPDR	55.24	9.00	
Severe NPDR	50.68	11.23	
PDR	55.24	8.57	
Control	49.06	10.90	

The study found no significant difference in the prevalence of PDR between sexes. However, males tended to have higher percentages and count in mild, moderate, and severe NPDR groups, while females had a higher percentage and count in the no DR and control groups compared to males. CCT tended to be higher in the earlier stages of DR and somewhat lower in the more advanced stages, but these variations were not statistically significant (Table 2).

**Table 2:** Variations of CCT with stages of DR DR: diabetic retinopathy; NPDR: non-proliferative diabetic retinopathy; PDR: proliferative diabetic retinopathy; CCT: central corneal thickness; RE: right eye; LE: left eye

CCT	Grades of Retinopathy	Number	CCT	Std. Deviation	P-value
CCT-RE	No DR	34	524.35	37.608	0.29
	Mild NPDR	34	525.38	30.498	
	Moderate NPDR	34	510.03	39.957	
	Severe DR	34	521.68	37.230	
	PDR	34	513.18	32.552	
	Control	34	512.15	33.522	
CCT-LE	No DR	34	524.88	36.214	0.298
	Mild NPDR	34	526.68	31.096	
	Moderate NPDR	34	514.00	42.857	
	Severe DR	34	527.94	41.262	
	PDR	34	517.53	37.946	
	Control	34	511.06	33.965	

The study also found that changes in corneal curvature, as indicated by keratometry values, were more pronounced in the proliferative stage of DR, with a statistically significant increase in k2 values of the left eye in the PDR group (Table 3).

**Table 3:** Variations of keratometry with stages of DR DR: diabetic retinopathy; NPDR: non-proliferative diabetic retinopathy; PDR: proliferative diabetic retinopathy; CCT: central corneal thickness; RE: right eye; LE: left eye; K1: horizontal keratometry values; K2: vertical keratometry values

Keratometry	Grades of Diabetic Retinopathy	No.	Mean	Std. Deviation	P-value
K1-RE	No DR	34	43.8685	1.31451	0.222
	Mild NPDR	34	43.9350	1.66118	
	Moderate NPDR	34	44.0109	1.40613	
	Severe NPDR	34	43.7882	1.58657	
	PDR	34	44.7206	2.20331	
	Control	34	43.8956	1.78462	
K2-RE	No DR	34	44.5147	1.35134	0.103
	Mild NPDR	34	44.8532	1.71610	
	Moderate NPDR	34	44.8247	1.31066	
	Severe NPDR	34	44.6188	1.58028	
	PDR	34	45.5915	1.97769	
	Control	34	44.7888	1.69297	
K1-LE	No DR	34	43.7209	1.26477	0.22
	Mild NPDR	34	44.0121	1.76915	
	Moderate NPDR	34	44.1318	1.40790	
	Severe NPDR	34	43.7844	1.69710	
	PDR	34	43.7844	1.69710	
	Control	34	44.2915	1.68519	
K2-LE	No DR	34	44.4329	1.31271	0.025
	Mild NPDR	34	44.9762	1.69554	
	Moderate NPDR	34	44.9703	1.30002	
	Severe NPDR	34	44.4774	1.55669	
	PDR	34	45.6565	1.90315	
	Control	34	44.8271	1.70456	

The mean HbA1c levels were relatively similar across different categories of DR, ranging from 7.87 to 8.25, with no statistically significant difference across grades of retinopathy. The standard deviations indicated variability within each category, with the highest variability seen in the no DR and mild NPDR categories (Table 4).

**Table 4:** Variations of HbA1c with stages of DR DR: diabetic retinopathy; NPDR: non-proliferative diabetic retinopathy

Grade of Diabetic Retinopathy	Mean HbA1c	Std. Deviation	P-value
No DR	8.23	2.51	0.942
Mild NPDR	7.87	2.41	
Moderate NPDR	8.17	1.89	
Severe NPDR	8.05	1.62	

The study concluded that there was no statistically significant relationship between CCT and keratometry values of both eyes with HbA1c levels (Table 5).

**Table 5:** Relationship between CCT, keratometry and HbA1c CCT: central corneal thickness; RE: right eye; LE: left eye; K1: horizontal keratometry values; K2: vertical keratometry values

		HbA1C
CCT-RE	Correlation	-0.027
	P-value	0.725
CCT-LE	Correlation	-0.065
	P-value	0.399
K1-RE	Correlation	-0.038
	P-value	0.627
K2-RE	Correlation	-0.099
	P-value	0.200
K1-LE	Correlation	-0.040
	P-value	0.603
K2-LE	Correlation	-0.078
	P-value	0.309

Overall, the study provides insights into the correlations between diabetes and ocular parameters, suggesting that while there are trends and relationships, many of the observed differences are not statistically significant, indicating a need for further research to better understand these associations.

## Discussion

This research observed a significant trend (p-value of 0.036) indicating that individuals with any form of DR (non-proliferative or proliferative) were generally older compared to those without retinopathy or the control group. Nargis also noted a direct link between age and the severity of DR (p-value 0.731) [3], whereas Klein and Klein did not find a significant link between age and the severity of DR [6]. Some studies, like Gadkari et al. (p=0.007) [7] and Machado et al. [8], observed a higher incidence of DR in males than females. However, this study did not reveal a significant difference in the prevalence of PDR between genders (p=0.627), although males were more likely to be found in the mild, moderate, and severe NPDR groups, whereas females were more prevalent in the no DR and control groups. The findings in this study indicate that individuals with DR generally possess a higher CCT compared to the control group, particularly in the earlier stages of the disease (no DR and mild NPDR).

However, as the severity of DR increases, the CCT tends to decrease slightly in moderate, severe NPDR, and PDR stages. The study notes that the variations in CCT across these groups were not statistically significant, with a p-value of 0.29, suggesting that while there are observable trends, they may not be clinically relevant. While some studies, such as Nargis [3], found a positive correlation between CCT and the severity of DR, other studies like Kaur et al. [4] did not observe a significant difference in CCT across different stages of DR. Tirkey et al.'s study [9] shows a significant correlation exists between increased CCT and the severity of DR and also with the duration of diabetes. Patients with thicker corneas are more likely to be in advanced stages of the disease. He also advised measuring CCT in diabetic patients for pre-surgery evaluations, assessing donor tissues, and monitoring conditions like glaucoma. Zengin et al. [10] in their study showed that the severity of retinopathy did not impact CCT. However, patients with mild to moderate NPDR had significantly higher CCT values compared to diabetic patients without retinopathy. Our study also looked into the relationship between the stages of DR and the measurements of keratometry (K1 and K2). It was observed that there was a statistically significant increase in K2 measurements in the left eye of individuals with PDR (p=0.025), suggesting that changes in corneal curvature might be more pronounced in the proliferative phase of DR.

Zengin et al. [10] in their study compared the mean steep keratometry across the subgroups of DR and found no significant difference (p-value = 0.4). Similarly, mean flat keratometry was not significantly different. Huseynova et al. [11] in 2017 studied the impact of DM on various corneal parameters using a Scheimpflug camera. The study found no statistically significant difference in the mean steep and flat keratometric values among different subgroups of diabetic patients (p-value = 0.469 and p-value = 0.423, respectively). This study did not uncover a significant link between CCT and keratometry measurements with HbA1c levels. In contrast, some studies, like Tirkey et al. [9] and Zengin et al. [10], reported that higher HbA1c levels were associated with increased corneal thickness, although the exact nature of this relationship requires further investigation over time. This study also failed to identify a statistically significant difference in average HbA1c levels across different stages of retinopathy. Similarly, Machado et al. [8] in their study found no statistically significant difference in the mean HbA1c levels across the varying grades of severity of DR. Conversely, the study by Nargis [3] established a strong positive correlation between HbA1c levels and the severity of DR (p-value = 0.01, r=0.887), and the study by Kaur et al. [4] demonstrated a significant correlation (p<0.05) between higher HbA1c levels and increased prevalence and severity of DR. The relationship between HbA1c levels and the severity of DR is complex and warrants careful examination. Findings in our study suggest that while there is a general trend of increasing HbA1c levels with the progression of DR, the relationship is not strictly linear. It was noticed that the highest HbA1c levels are observed not only in patients with advanced stages of retinopathy but also in those with no retinopathy at all. This finding challenges the assumption that higher HbA1c levels directly correlate with the severity of DR. It underscores the need for a nuanced understanding of the interplay between glycemic control and retinal health. Factors such as the duration of diabetes, individual patient variability, and other comorbid conditions may also influence these outcomes. Therefore, while HbA1c remains a critical marker for diabetes management, its role in predicting the severity of DR is multifaceted and requires further research to elucidate the underlying mechanisms.

However, the small number of participants and the short duration of our study limits the conclusions that can be drawn. A small sample size may affect the representativeness and reliability of the results, by affecting the statistical power and significance in the subgroup analysis. Since the study is limited to a specific population, the conclusions lack generalizability. Future studies in a broader range of racial and geographic populations would help improve the generalizability of the conclusions. Further studies need to be carried out including a wide racial population, which is lacking in our study. This warrants a further long-term study to assess the effects of diabetes on corneal thickness and keratometry. As our study is cross-sectional, the most recent HbA1c reports were available with the patient at the time of his/her presentation to our OPD. Previous reports were not available with all patients. Some patients were started on oral hypoglycemics and later shifted to insulin. However, incomplete and varied data were available regarding the patients’ usual glycemic control and the consistency of antidiabetic medication use. These factors could influence the relationship between HbA1c levels and retinopathy thereby affecting the results. Similarly, the distribution of retinopathy is not equal across stages. However, in order to study if CCT and keratometry vary across the different stages of DR, we have taken an equal number of patients in each group. This may introduce a bias. The research is constrained by its single-center approach and limited participant pool and small sample size. Furthermore, it failed to establish a meaningful connection between CCT, keratometry readings, and HbA1c levels. Conversely, other studies have indicated that elevated HbA1c levels may correlate with greater corneal thickness, yet the precise dynamics of this association necessitate additional longitudinal research. To negate this limitation of our study, we intend to carry on a further prospective longitudinal study. This would give a better generalization of diabetic effects on corneal, refractive and ocular interventions (both intra and extraocular surgeries).

## Conclusions

The research revealed several key findings about the relationship between diabetes and eye health. Like many other studies carried out in the past, we too found a progression of DR with age. Age was seen to play a major role in the progression of DR, with older individuals more likely to have severe forms. So, while there were some clear patterns, especially concerning age and gender, most variations in eye health measures, such as HbA1c, CCT, and keratometry, were not statistically significant, suggesting that other factors might have a role to play. However, advanced stages of DR showed some significance in the changes in K2 values, indicating changes in the shape of the cornea. We therefore intend to study further over a longer duration including a larger spectrum of diabetic patients.
